# Hypo-attenuated leaflet thickening in surgically-implanted mitral bioprosthesis

**DOI:** 10.1186/s13019-020-01120-3

**Published:** 2020-05-07

**Authors:** Soh Hosoba, Makoto Mori, Yoshihiro Goto, Yuichiro Fukumoto, Tetsuro Shimura, Masanori Yamamoto

**Affiliations:** 1 Division of Cardiovascular Surgery, Toyohashi Heart Center, 21 Gofuntori, Oyamacho, Toyohashi, Aichi 4418530 Japan; 2 Section of Cardiac Surgery, Yale University School of Medicine, New Haven, CT USA; 3 Division of Cardiology, Toyohashi Heart Center, Toyohashi, Japan

**Keywords:** Mitral valve, Hypo-attenuated leaflet thickening, Leaflet thrombosis

## Abstract

**Background:**

Hypo-attenuated leaflet thickening (HALT) in bioprosthetic aortic valve has been studied, but its equivalent in bioprosthetic mitral valve (bMV) remains uncharacterized. We sought to identify the prevalence, hemodynamic characteristics, and significance of anticoagulation therapy in bMV HALT.

**Methods:**

A single-center cross-sectional study of 53 consecutive patients who underwent mitral valve replacement (MVR) with bMV between 2007 and 2017 was conducted. Cardiac-gated contrasted CT scans were obtained. Anticoagulant and antiplatelet therapy use were ascertained at the time of hospital discharge and CT scanning. Patient characteristics, postoperative stroke, and hemodynamic profile by echocardiogram were obtained to descriptively characterize the prevalence and characteristics associated with bMV HALT.

**Results:**

Three patients (5.7%) were found to have a HALT on bMV. The mean time from index MVR to CT scan was 3.4 ± 0.8 years in HALT cohort and 3.4 ± 2.7 years in non-HALT cohort. Fifty patients (94.3%) were discharged on warfarin, and 37 patients (69.8%) were on warfarin at the time of CT scans. One patient with HALT was on therapeutic warfarin at the time of the CT scan that identified HALT. All three patients were asymptomatic at the time of CT scan. In patients with HALT, mean transmitral pressure gradient were 8, 5, and 2.7 mmHg, all with trivial or mild mitral regurgitation.

**Conclusions:**

In this study, the prevalence of HALT was low at 5.7%, all presenting without symptoms. One patient presented with HALT while on therapeutic oral anticoagulation, which may suggest thrombotic etiology may not adequately explain HALT.

## Key question

Does hypo-attenuated leaflet thrombosis occur in bioprosthetic mitral valve?

## Key finding

Hypo-attenuated leaflet thrombosis was observed in patients who underwent bioprosthetic mitral valve replacement.

## Take-home message

Although rare, hypo-attenuated leaflet thrombosis of mitral valve occurs and must be further characterized.

## Introduction

A series of reduced aortic-valve leaflet motion and a radiographic finding of hypoattenuating opacities in bioprosthetic aortic valve was first described in 2015 [1], bringing attention to this previously unrecognized entity. Using 4D volume-rendered computed tomography (CT) scans, this was identified in 40% of the evaluated cohort, occurring in both transcatheter and surgically implanted aortic valve prostheses. Recent analysis of two large registries reported 13% prevalence of this entity, and reported a higher prevalence in valves implanted via transcatheter aortic valve replacement (TAVR) compared to those implanted via surgical aortic valve replacement (SAVR) [2]. Importantly, its previous perception as a benign finding was questioned by the demonstration of increased incidences of cerebrovascular events associated with subclinical leaflet thrombosis in this series [2].

Hypo-attenuated leaflet thickening (HALT) of bioprosthetic valve is a hallmark finding of subclinical leaflet thrombosis [3]. Its characteristics have been described fairly extensively in aortic valves, but its occurrence and characteristics are unknown in bioprosthetic mitral valves (bMV). Mitral valve prosthesis may be at higher risk of leaflet thrombosis for its exposure to lower flow compared to that of prostheses in the aortic position. In addition, possible impact of anticoagulation strategy on HALT would be of interest in the context of current variability in anticoagulation following mitral valve replacement [4] despite the guideline recommendation [5]. This study describes the characteristics and prevalence of HALT in surgically-implanted bMV in a single-center series.

## Materials and methods

### Patient selection

A cross-sectional study of 175 consecutive patients who underwent mitral valve replacements with bMV at Toyohashi Heart Center, Japan, between 2007 and 2017 was conducted. Fifty-six patients died during follow-up. Thirty-four patients were excluded for history of chronic kidney disease, due to an elevated risk of further renal injury from contrasted CT scan. Eighty-five surviving patients without chronic kidney disease were contacted between June and October 2017 to undergo contrasted multi-dimensional CT scan, of whom 53 agreed to undergo the scan between June and October 2017 to evaluate the present of HALT in bMV (Additional file 1: Figure S1). Toyohashi Heart Center Institutional Review Board approved this study, and individual patient consent was obtained at the time of CT scanning. Anticoagulant and antiplatelet therapy use were ascertained at the time of hospital discharge from index hospitalization and at the time of CT scanning. At our center, patients undergoing MVR with bMV are discharged on warfarin and 81 mg aspirin unless contraindicated, and anticoagulation is continued for up to 3 months, after which anticoagulation is discontinued unless there are other indications to continue.

### CT scanning and HALT definition

All patients underwent cardiac-gated contrasted CT scan using a 256-slice CT scanner (Brilliance iCT; Philips Medical Systems, Eindhoven, the Netherlands) at the Toyohashi Heart Center. Cardiac gating was performed with prospective electrocardiogram triggering (75% of R-R interval) to obtain a slice thickness of 2.5 mm. The scan was performed between the tracheal bifurcation and the diaphragm with the following parameters: collimation width, 32 × 0.625 mm; rotation time, 330 ms/revolution; tube voltage, 120 kV; and maximum effective tube current, 412 mA. Image reconstruction was gated prospectively to 30–45% of R-R interval. CT images were reconstructed across the entire cardiac cycle using a cardiac standard filter with a slice thickness of 2.5 mm. CT datasets were transferred to an offline workstation (Intelli Space Portal; Philips Medical Systems) for image analysis. The images were evaluated by an attending imaging cardiologist (T.S) and an attending cardiac surgeon and consensus was obtained in all findings of HALT.

HALT was defined as the presence of a low-density area on bMV during systole on image with the most closure of the valve, which parallels the definition of HALT in aortic valve bioprosthesis [3]. Transthoracic echocardiogram was obtained at the time of CT scan. Data were collected on patient demographics, comorbidity, presenting symptoms, operative details, postoperative survival and stroke, echocardiographic and CT scan findings, and use of anticoagulant or antiplatelet therapy at the time of index hospital discharge and at the time of CT scans. CT characteristics and echocardiographic findings including ejection fraction (EF), mitral regurgitation (MR), mean mitral pressure gradient (PG), and mitral valve area (MVA) are reported. The prevalence of HALT was calculated by the number of patients with HALT divided by the 53 patients who underwent CT scan as a denominator. All analysis was conducted using SAS version 9.4 (SAS Institute, Cary NC).

## Results

Of the 53 patients who underwent CT evaluation, 3 patients (5.7%) were found to have a HALT on bMV, each affecting different leaflets. The mean time from index MVR to CT scan was 3.4 ± 0.8 years in HALT cohort and 3.4 ± 2.7 years in non-HALT cohort. Table 1 summarizes patient characteristics of the entire cohort. Oral anticoagulant and antiplatelet therapy use at the time of hospital discharge and at the time of CT scan are summarized in Table 2. Among the entire cohort of 53 patients, 74% were discharged on the dual therapy of single antiplatelet agent (SAPT) and warfarin, and additional 21% were on warfarin only. At the time of CT scan, 43% were on warfarin only, and 26% were on the dual therapy of SAPT and warfarin. One patient who was found to have HALT was on warfarin with the INR in the therapeutic range at the time of CT scan.

**Table 1:** Baseline characteristics of patients with CT scan

Variables	***N*** = 53
Age (years)	71.6 ± 8.9
Male	26 (49.0)
Diabetes	12 (22.6)
COPD	0 (0.0)
Creatinine (mg/dL)	1.02 ± 0.95
Chronic kidney disease	0 (0.0)
NYHA functional class I or II	42 (79.2)
NYHA functional class III or IV	11 (20.8)
Previous PMI	0 (0.0)
Previous CABG	1 (1.9)
Previous valve surgery	4 (7.5)
Operative variables	
Emergency	3 (5.7)
Urgent	0 (0.0)
Elective	50 (94.3)
Concomitant operations	
MAZE	34 (64.2)
CABG	9 (17.0)
AVR	14 (26.4)
TAP	29 (54.7)
Cross-clamp time (minutes)	105.7 ± 31.2
Perfusion time (minutes)	154.4 ± 37.5
Operation time (minutes)	271.5 ± 54.0
Valve size (mm)	
25	15 (28.3)
27	27 (50.9)
29	10 (18.9)
31	1 (1.9)
Valve type	
Carpentier-Edwards Pericardial Bioprosthesis	12 (22.6)
Magna Ease	16 (30.2)
Mosaic	15 (28.3)
St. Jude Medical Epic	10 (18.9)
Stroke within 30 days of operation	2 (3.8)

Data are displayed as mean ± SD or n (%). *AVR* Aortic valve replacement, *CABG* Coronary artery bypass grafting, *COPD* Chronic obstructive pulmonary disease, *NYHA* New York Heart Association, *PMI* Pacemaker implantation, *TAP* Tricuspid annuloplasty

**Table 2:** Oral anticoagulant and antiplatelet therapy use at the time of hospital discharge and at the time of CT scan

Medications	At discharge (N = 53)	At CT (N = 53)
SAPT	3 (5.7)	13 (24.5)
DAPT	0 (0.0)	2 (3.8)
SAPT + warfarin	39 (73.6)	14 (26.4)
SAPT + DOAC	0 (0.0)	0 (0.0)
Warfarin	11 (20.8)	23 (43.4)
DOAC	0 (0.0)	1 (1.9)
Unknown	0 (0.0)	0 (0.0)

Data are displayed as n (%). *CT* Computed tomography, *SAPT* Single antiplatelet therapy, *DAPT* Dual antiplatelet therapy, *DOAC* Direct oral anticoagulant

**Fig. 1:**
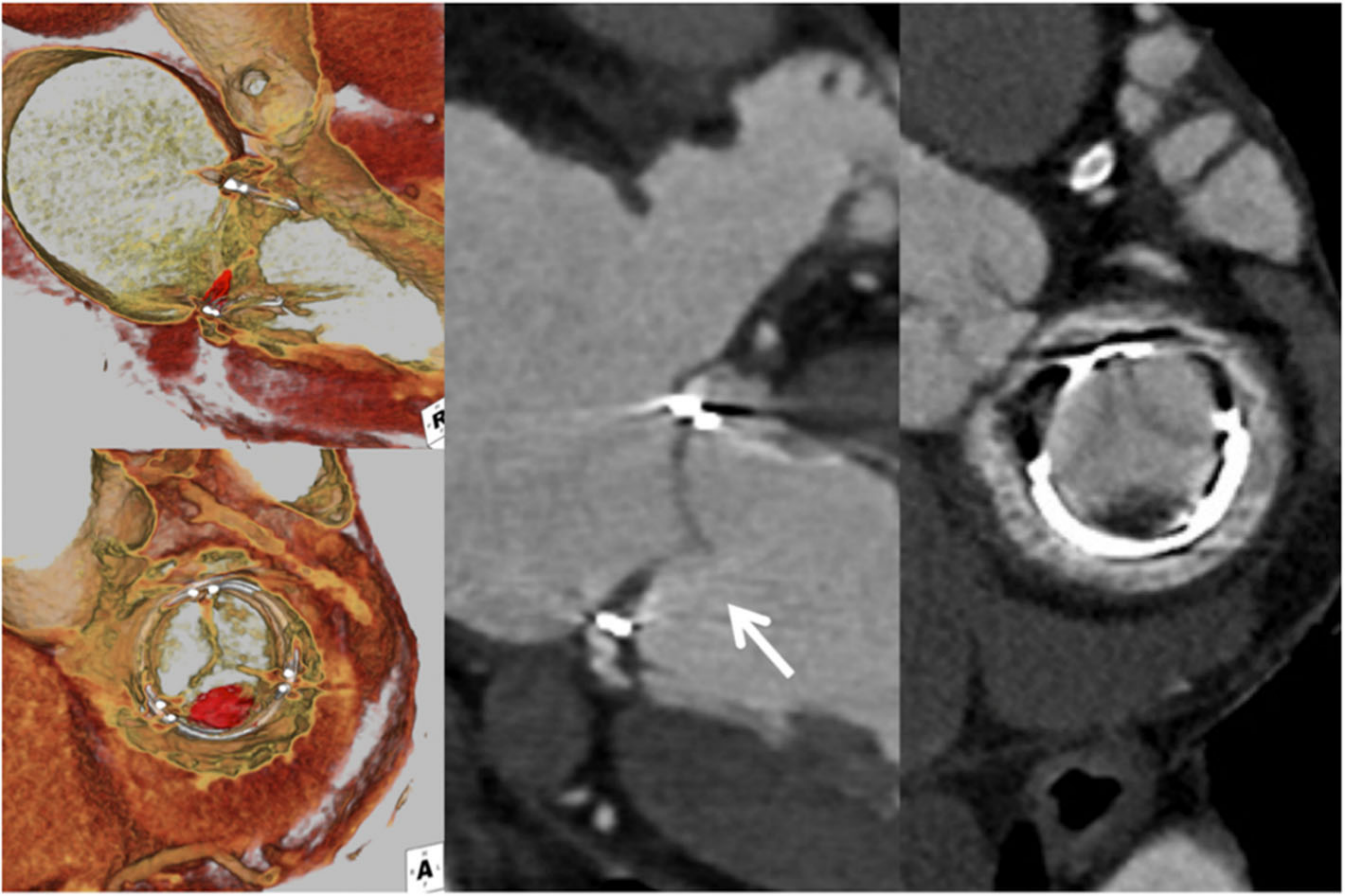
65-year-old male. 3.6 years out of mitral valve replacement. Long-axis and short-axis view. CT image demonstrated a HALT in one leaflet on the periphery near the sewing ring. All panels were obtained on the same day

**Fig. 2:**
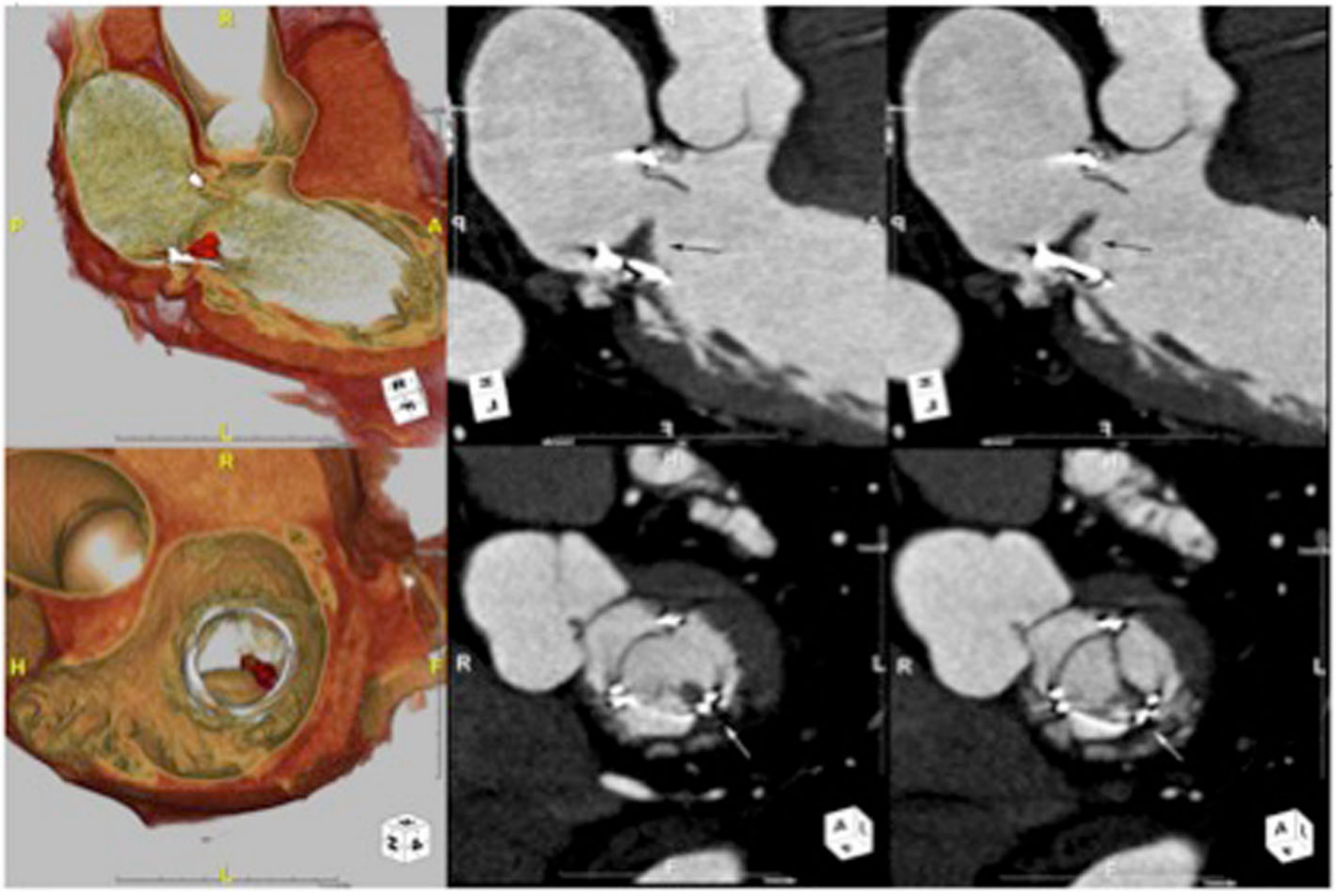
74-year-old female. 2.3 years out of mitral valve replacement. CT image demonstrated a HALT in a posteromedial single leaflet

**Fig. 3:**
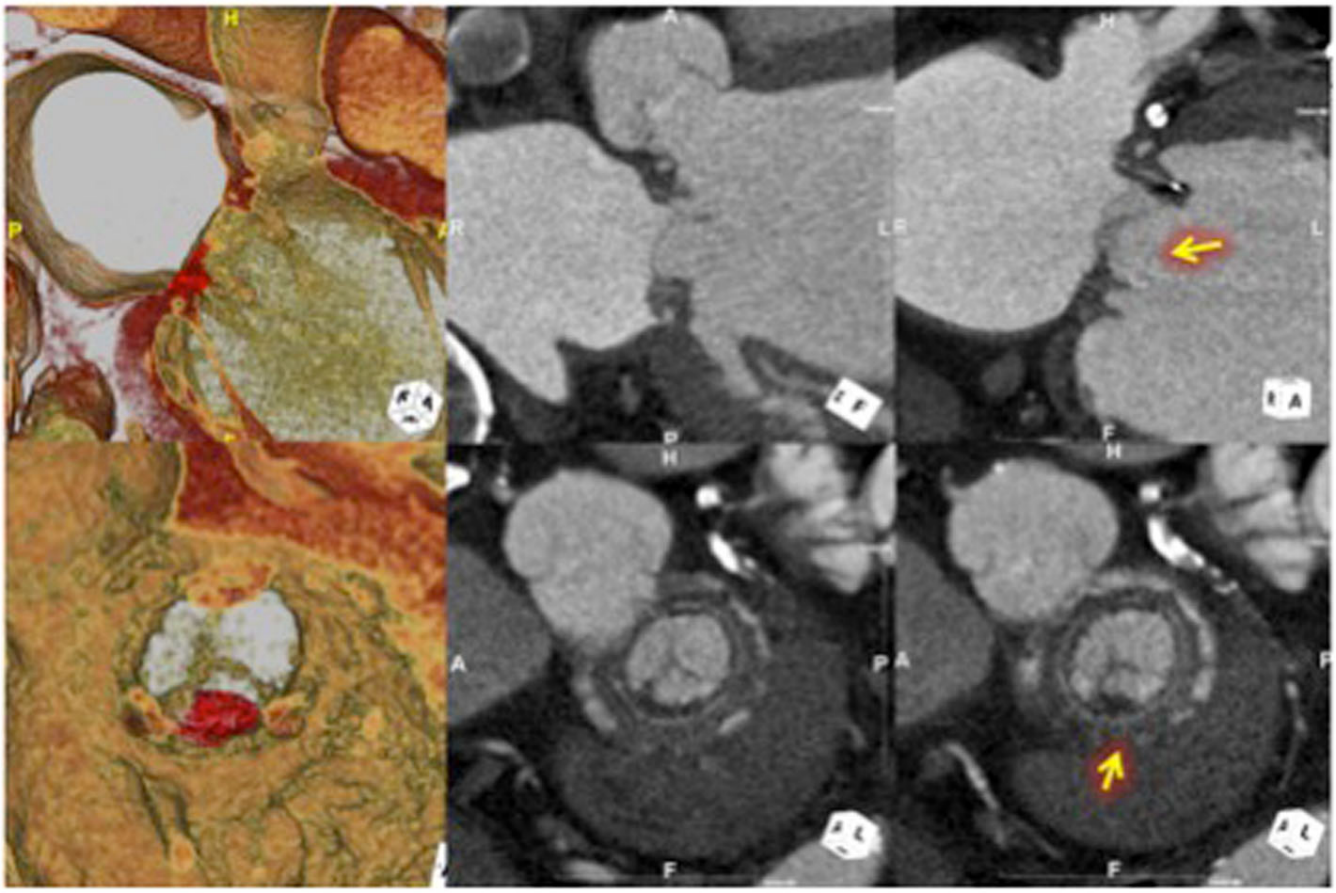
72-year-old male. 4 years out of mitral and aortic valve replacement. CT showed a HALT extending to almost the entire leaflet

All three patients were asymptomatic at the time of CT scan. Table 3 summarizes pertinent finding in each patient who was found to have HALT. Figure 1 displays CT findings of HALT in the three patients. First patient was 3.6 years out of mitral valve replacement (MVR) with 27 mm Magna Ease bMV (Carpentier-Edwards Lifescience, Irvine CA). CT image demonstrated a HALT in one leaflet on the periphery near the sewing ring (Fig. 1). Echocardiogram demonstrated EF of 45%, trace MR with mean PG of 8 mmHg, and MVA of 2.24 cm^2^ at the time of CT. Second patient was 2.3 years out of MVR. The mitral bioprosthesis was 27 mm Mosaic bMV (Medtronic, Minneapolis, MN). CT image demonstrated a HALT in a posteromedial single leaflet (Fig. 2). Echocardiogram demonstrated EF of 61%, mild MR with PG of 5 mmHg with MVA of 2.1cm^2^. Third patient was 4 years out of MVR and concomitant AVR with 27 mm Magna Ease bMV. CT showed a HALT extending to almost the entire leaflet (Fig. 3). Echocardiogram demonstrated EF of 64%, mild MR with PG of 2.7 mmHg and MVA of 2.76 cm^2^.

**Table 3:** Summary of three patients with HALT

	Age (years)/Sex	Preoperative diagnosis	Procedure	Antithrombotic therapy at discharge	At the timing of detection	Degree of leaflet immobility in TTE (concordance with CT)	HALT area (mm^**2**^)	Clinical symptom	Max mean PG (mmHg) /MR
1	65/Male	PMR	MVR, CABG	Coumadin	Aspirin 3.6 years	Moderate	40.8	None	8/Trivial
2	74/Female	MS	Redo-MVR (previous ASD repair)	Coumadin and aspirin	Coumadin 2.3 years	Mild	40.3	None	5/Mild
3	72/Male	AS, MR	AVR, MVR, MAZE	Aspirin	None 4.1 years	Moderate	48.7	None	2.7/Mild

*AS* Aortic stenosis, *ASD* Atrial septal defect, *AVR* Aortic valve replacement, *CABG* Coronary artery bypass graft, *CT* Computed tomography, *MR* Mitral regurgitation, *HALT* Hypo-attenuated leaflet thickening, *MS* Mitral stenosis, *MVR* Mitral valve replacement, *PG* Pressure gradient, *PMR* Papillary muscle rupture, *TTE* Transthoracic echocardiogram

**Fig. 4:**
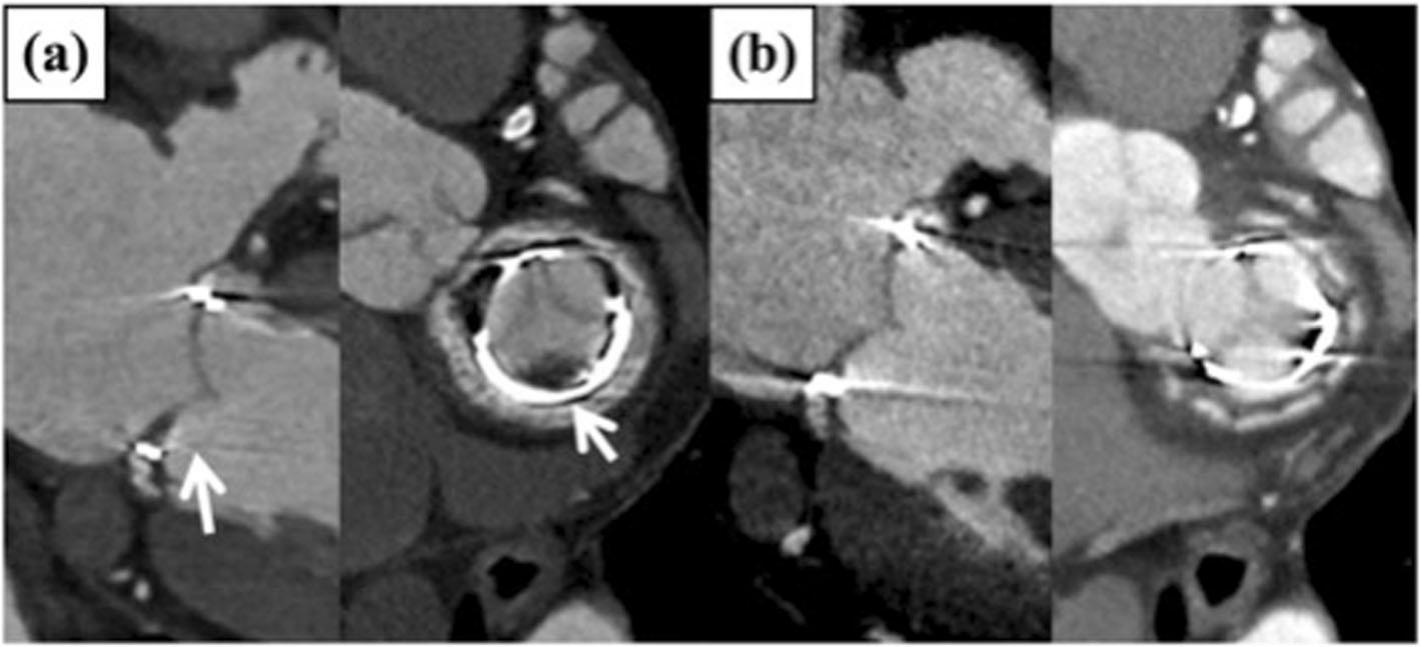
**a** initial CT in 65-year-old male. **b** Repeat CT scan was obtained 12 months after prasugrel and aspirin, and demonstrated complete resolution of the thrombus

First patient had stable angina related to failed saphenous vein graft, 38 months postoperatively and underwent cardiac-gated CT scan. This CT scan also demonstrated HALT. He underwent percutaneous coronary intervention (PCI) for the vein graft with resolution of symptoms. Dual antiplatelet therapy was initiated following the PCI. Repeat CT scan was obtained 12 months after the event to reevaluate coronary and mitral valve, and demonstrated complete resolution of the thrombus (Fig. 4). His mean transvalvuar pressure decreased to 5 mmHg from 8 mmHg at the time of first CT.

## Discussion

In this single-center cross-sectional study, the prevalence of HALT in bMV was low at 5.7%. This number should be interpreted in the context of the anticoagulant use pattern, which was routine anticoagulation with warfarin along with 81 mg aspirin for at least 3 months following the index operation. Surprisingly, one patient was on warfarin with the INR in the therapeutic range at the time of the CT scan, in which HALT was identified. Possible explanations are numerous, and include the possibility that HALT is a point in the spectrum of structural valve degeneration, in which the etiology is not necessarily a thrombus formation [6]. All three patients with HALT presented without valve-related symptoms and echocardiographic abnormality was identified in all three patients. The extent of hypoattenuation varied from those only occupying minimal portion of the leaflet to the one extending to almost the entire leaflet. The temporal progression and potential reversal with anticoagulation remains topic of further investigation. This study offers the first insight into this previously suspected but poorly elucidated entity. Its association with the longevity of prosthetic valve is of interest as well.

In the aortic valve domain, majority of patients with HALT detected by multi-detector CT (MDCT) appears to have normal echocardiographic findings. In a series of 140 patients who underwent TAVR with Edwards Sapien XT, MDCT detected HALT in 5 patients (4%), although 4 of them were asymptomatic with normal echocardiographic findings [7]. Similar pattern was expected in HALT in bMV, and this prompted us to obtain both MDCT and echocardiographic imaging in our cohort. Indeed, of the three patients with HALT detected on MDCT, echocardiographic findings were abnormal in all three patients. The incidence of HALT in bioprosthetic aortic valve varies across studies but most resides in the range between 4 and 12% [2, 7–9]. The incidence of 5.7% in our bMV cohort falls within this range. The original report by Makkar and colleagues is an outlier with the prevalence of 40% at 30 days in their series of 55 patients who underwent either SAVR or TAVR [1]. The vast majority of the patients in Makkar’s study was only on either dual or single antiplatelet therapy, which may partly explain the discrepancy in the prevalence. However, it should also be noted that in a series of 249 Sapien S3 TAVR patients who were only on dual or single antiplatelet therapy, the prevalence of HALT was 10% [9]. Importantly, this study also reported that CT finding of HALT resolved almost completely following full anticoagulation [9], corroborating its hypothesized thrombotic etiology. Unfortunately, repeat CT scans following the institution of anticoagulation in patients with HALT were not obtained in our cohort.

Structural valve degeneration (SVD) and valve longevity remains active area of investigation with limited long-term data compared to the surgical aortic valve prosthesis. A standardized definition of SVD was proposed in 2018 to help facilitate this [10]. HALT and leaflet thrombosis are suspected to play a role in accelerating SVD, possibly via triggering inflammation and subsequent fibrocalcific remodeling of valve leaflets [6]. Subclinical leaflet thrombosis in bioprosthetic aortic valve appears to be more common in TAVR valves compared surgically placed valves [2], with balloon-expandable valve being associated with highest risk of overt valve thrombosis in TAVR [11]. In vitro study has suggested that the difference among valves in thrombogenicity is due to the flow properties [12]. The trans-mitral flow property differs significantly from that of trans-aortic flow, which may produce a differential effect towards the thrombogenicity. The prevalence of HALT in our series was relatively low compared to previously reported prevalence in aortic valve bioprosthesis, although this is difficult to interpret in the context of limited sample size.

In the aortic valve domain, it is hypothesized that HALT exist along the spectrum of SVD in that HALT represents the earliest phase of SVD, with a more severe form manifesting in reduced leaflet motion and hemodynamically overt valve thrombosis, eventually leading to premature SVD [6, 10]. Extrapolating from the excellent long-term durability of surgically implanted aortic valves [13] and a differential in the risk of HALT between SAVR and TAVR valves, the sequence of events likely occurs more commonly in TAVR valves compared to SAVR valves [2, 14]. In our series, all three patients with HALT in bMV had hemodynamically occult HALT with mean PG in a normal range with normal leaflet motions on echocardiogram. Importantly, the cumulative incidence of HALT increases as the duration of follow-up increase. In a series of 70 patients following TAVR, only 1 was found to have HALT at the time of discharge, 7 at 6-month follow-up, and 10 at 1-year [15]. Our cohort had a mean follow up time of 3.4 years since the index valve replacement, which yielded the HALT prevalence of 3 among 53 patients. Longer follow up would provide insights on temporal changes in the risk of HALT in bMV, although obtaining serial contrasted CT scans poses logistical challenges.

The finding of HALT has been associated with increased risk of transient ischemic attack (TIA) or stroke [2]. In the series of 890 patients who underwent surgical or transcatheter AVR, those with HALT had stroke or TIA rate of 7.9 per 100 person-years whereas those without HALT had the rate of 2.4 per 100 person-years, and this difference was statistically significant [2]. Interestingly, the presence of reduced leaflet motion alone was not associated with a different risk of cerebrovascular event. In our series, none of the three patients with HALT had neurologic symptoms, and brain imaging was deferred. Evaluation of the incidence of cerebrovascular event associated with bMV at a larger scale is in need. Current data are insufficient to infer whether routine screening for this entity should be recommended. Additionally, the role of lipid lowering agent is debated for it relationship with slowing of aortic valve degeneration. Only the first patient was on a lipid lowering agent at the time of presentation and its significance remain unclear.

Reversal of HALT and presumably the protective effect of anticoagulation are evident from previous observational studies. It also appears that anticoagulation with direct oral anticoagulant or warfarin is effective towards HALT while antiplatelet therapy alone may not be^2^. An analysis from the national Society of Thoracic Surgeons Adult Cardiac Surgery database indicated that the use of anticoagulation following bioprosthetic mitral valve replacement varies significantly across centers and surgeons [4], despite the class IIa recommendation according to the American Heart Association/American College of Cardiology guideline [16, 17]. In addition, the higher prevalence of atrial fibrillation in the population with mitral valve disease and subsequent need for anticoagulation may modulate the risk of HALT in bMV. This association is also currently unknown and warrants further investigation.

### Limitations

Aside from limitations associated with single-center retrospective design of this study, the small sample size precluded application of longitudinal models to account for the time-dependent nature of HALT risk. Therefore, we conducted cross-sectional analysis of prevalence.

## Conclusions

In this first series of HALT in surgically-implanted bMV, the prevalence of HALT evaluated via CT was low at 5.7%. All 3 patients found to have bMV HALT were asymptomatic. One patient presented with HALT while on therapeutic oral anticoagulation. Further study is needed to characterize the clinical significance of this finding in the mitral position.

## Supplementary information

**Additional file 1: Figure S1.** CONSORT-style diagram. Figure shows starting cohort and subsequent exclusions and reasons for exclusion to reach the final cohort.

## Data Availability

Supporting data are not available due to the sensitive nature of the data.

## References

[1] Makkar RR, Fontana G, Jilaihawi H, Chakravarty T, Kofoed KF, De Backer O (2015). Possible subclinical leaflet thrombosis in bioprosthetic aortic valves. N Engl J Med.

[2] Chakravarty T, Søndergaard L, Friedman J, De Backer O, Berman D, Kofoed KF (2017). Subclinical leaflet thrombosis in surgical and transcatheter bioprosthetic aortic valves: an observational study. Lancet..

[3] Jilaihawi H, Asch FM, Manasse E, Ruiz CE, Jelnin V, Kashif M (2017). Systematic CT methodology for the evaluation of subclinical leaflet thrombosis. JACC Cardiovasc Imaging.

[4] Schwann TA, Habib RH, Suri RM, Brennan JM, He X, Thourani VH (2016). Variation in warfarin use at hospital discharge after isolated bioprosthetic mitral valve replacement: an analysis of the Society of Thoracic Surgeons adult cardiac surgery database. Chest..

[5] Nishimura RA, Otto CM, Bonow RO, Carabello BA, Erwin JP, Guyton RA (2014). 2014 AHA/ACC guideline for the management of patients with valvular heart disease: a report of the American College of Cardiology/American Heart Association task force on practice guidelines. J Thorac Cardiovasc Surg.

[6] Rodriguez-Gabella T, Voisine P, Puri R, Pibarot P, Rodes-Cabau J (2017). Aortic bioprosthetic valve durability: incidence, mechanisms, predictors, and Management of Surgical and Transcatheter Valve Degeneration. J Am Coll Cardiol.

[7] Leetmaa T, Hansson NC, Leipsic J, Jensen K, Poulsen SH, Andersen HR (2015). Early aortic transcatheter heart valve thrombosis: diagnostic value of contrast-enhanced multidetector computed tomography. Circ Cardiovasc Interv.

[8] Hansson NC, Grove EL, Andersen HR, Leipsic J, Mathiassen ON, Jensen JM (2016). Transcatheter aortic valve thrombosis: incidence, predisposing factors, and clinical implications. J Am Coll Cardiol.

[9] Pache G, Schoechlin S, Blanke P, Dorfs S, Jander N, Arepalli CD (2016). Early hypo-attenuated leaflet thickening in balloon-expandable transcatheter aortic heart valves. Eur Heart J.

[10] Dvir D, Bourguignon T, Otto CM, Hahn RT, Rosenhek R, Webb JG (2018). Standardized definition of structural valve degeneration for surgical and Transcatheter bioprosthetic aortic valves. Circulation..

[11] Jose J, Sulimov DS, El-Mawardy M, Sato T, Allali A, Holy EW (2017). Clinical bioprosthetic heart valve thrombosis after Transcatheter aortic valve replacement: incidence, characteristics, and treatment outcomes. JACC Cardiovasc Interv.

[12] Richardt D, Haban-Rackebrandt SL, Stock S, Scharfschwerdt M, Sievers HH (2018). A matter of thrombosis: different thrombus-like formations in balloon-expandable transcatheter aortic valve prostheses. Eur J Cardiothorac Surg.

[13] Zibdeh O, Bugg I, Patel S, Twine G, Unsworth-White J (2018). Randomized trial of the Carpentier-Edwards supra-annular prosthesis versus the Medtronic mosaic aortic prosthesis: 10-year results. Eur J Cardiothorac Surg.

[14] De Marchena E, Mesa J, Pomenti S, Marin Y, Kall C, Marincic X (2015). Thrombus formation following transcatheter aortic valve replacement. JACC Cardiovasc Interv.

[15] Yanagisawa R, Hayashida K, Yamada Y, Tanaka M, Yashima F, Inohara T (2017). Incidence, predictors, and mid-term outcomes of possible leaflet thrombosis after TAVR. JACC Cardiovasc Imaging.

[16] Nishimura RA, Otto CM, Bonow RO, Carabello BA, Erwin JP, Fleisher LA (2017). 2017 AHA/ACC focused update of the 2014 AHA/ACC guideline for the Management of Patients with Valvular heart disease: a report of the American College of Cardiology/American Heart Association task force on clinical practice guidelines. J Am Coll Cardiol.

[17] Nishimura RA, Otto CM, Bonow RO, Carabello BA, Erwin JP, Guyton RA (2014). 2014 AHA/ACC guideline for the Management of Patients with Valvular heart disease: executive summary: a report of the American College of Cardiology/American Heart Association task force on practice guidelines. J Am Coll Cardiol.

