# Supplementary figures and images for: Hypo-attenuated leaflet thickening in surgically-implanted mitral bioprosthesis
Source: J Cardiothorac Surg. 2020 May 7;15:74. doi: 10.1186/s13019-020-01120-3 (PMC7206689; doi:10.1186/s13019-020-01120-3)

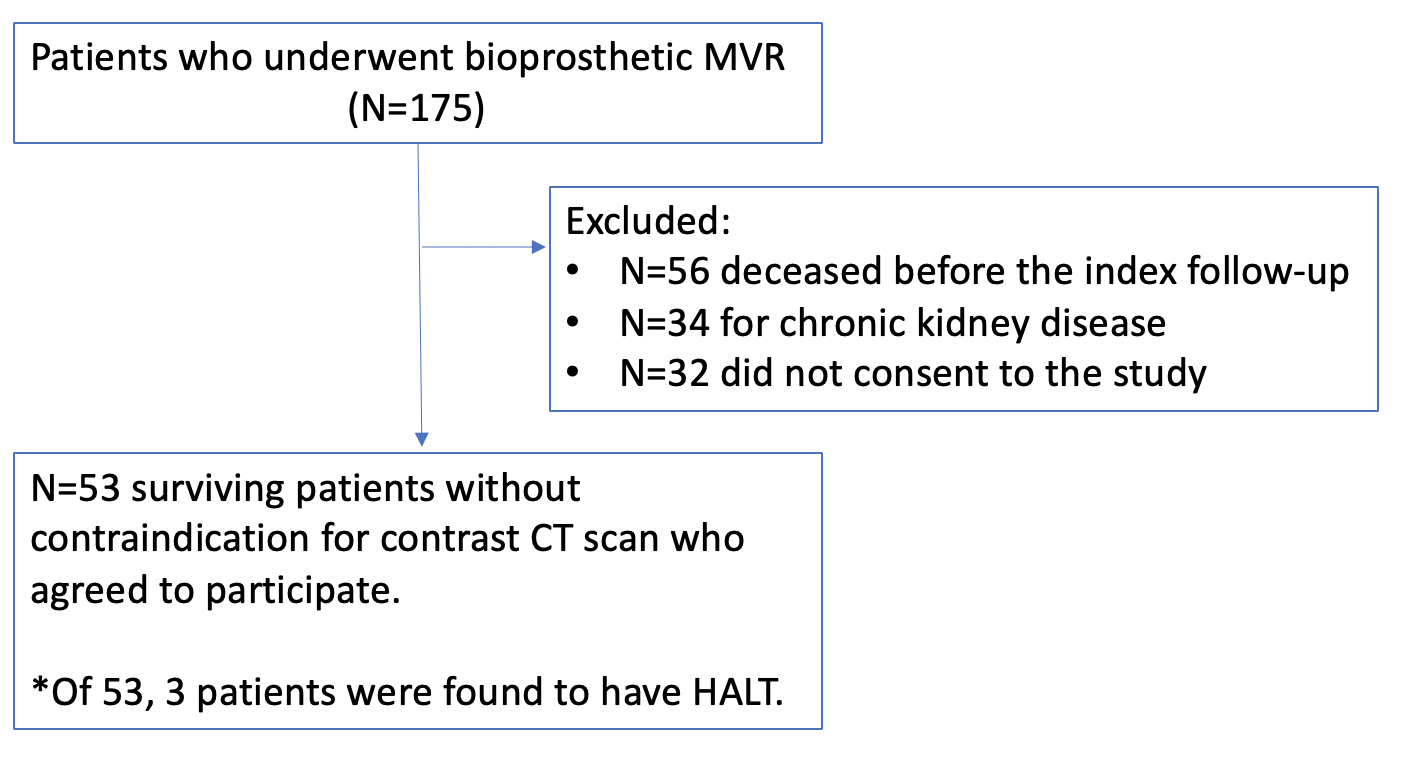

Supplement: Supplementary file 1 — Additional file 1: Figure S1. CONSORT-style diagram. Figure shows starting cohort and subsequent exclusions and reasons for exclusion to reach the final cohort. [file 13019_2020_1120_MOESM1_ESM.png]
